# Acquisition of Androgen Independence by Human Prostate Epithelial Cells during Arsenic-Induced Malignant Transformation

**DOI:** 10.1289/ehp.7832

**Published:** 2005-05-05

**Authors:** Lamia Benbrahim-Tallaa, Mukta M. Webber, Michael P. Waalkes

**Affiliations:** 1 Inorganic Carcinogenesis Section, Laboratory of Comparative Carcinogenesis, National Cancer Institute at the National Institute of Environmental Health Sciences, National Institutes of Health, Department of Health and Human Services, Research Triangle Park, North Carolina, USA; 2 Department of Medicine, and; 3 Department of Zoology, Michigan State University, East Lansing, Michigan, USA

**Keywords:** androgen independent, AR, arsenic, cancer progression, malignant transformation, prostate

## Abstract

Lethal phenotypes of human prostate cancer are characterized by progression to androgen independence, although the mechanisms behind this progression remain unclear. Arsenic is a potential human prostate carcinogen that may affect tumor progression. In this study, we used a prostate cancer cell model in which an immortalized, nontumorigenic human prostate epithelial cell line (RWPE-1) had been malignantly transformed by chronic low-level arsenic to help determine whether arsenic affects prostate tumor progression. Control and CAsE-PE (chronic-arsenic–exposed human prostate epithelial) cells were continuously maintained in a complete medium [keratinocyte serum-free medium (K-SFM) with bovine pituitary extract and epidermal growth factor] or in a steroid-depleted medium (K-SFM alone). The arsenic-transformed cells showed a more rapid proliferation rate in complete medium than did control cells and also showed sustained proliferation in steroid-reduced medium. Although both control and CAsE-PE cells showed similar levels of androgen receptor (AR), androgens were less effective in stimulating cell proliferation and AR-related gene expression in CAsE-PE cells. For instance, dihydrotestosterone caused a 4.5-fold increase in prostate-specific antigen transcript in control cells but only a 1.5-fold increase in CAsE-PE cells. CAsE-PE cells also showed relatively low levels of growth stimulation by nonandrogen steroids, such as estradiol. Thus, arsenic-induced malignant transformation is associated with acquired androgen independence in human prostate cells. This acquired androgen independence was apparently not due to AR up-regulation, increased activity, or altered ligand specificity. The precise manner in which arsenic altered CAsE-PE growth and progression is undefined but may involve a bypass of AR involving direct stimulation of downstream signaling pathways.

The carcinogenicity of arsenic in humans has been unambiguously demonstrated in a variety of epidemiologic studies (Pott et al. 2001). Inorganic arsenic exposure has been associated with cancers of the skin, lung, liver, kidney, and urinary bladder [National Toxicology Program (NTP) 2000]. Although arsenic carcinogenesis has many other targets, a significant association has also been observed between prostate cancer and chronic arsenic exposure (Chen and Wang 1990; Lewis et al. 1999). Arsenic can cause malignant transformation of human prostate epithelial cells *in vitro*, and these CAsE-PE (chronic-arsenic–exposed prostate epithelial) cells produce aggressive, carcinoma-like tumors when inoculated into nude mice (Achanzar et al. 2002). There is also evidence that arsenic can enhance tumor progression. For instance, oral exposure to arsenic in mice not only increases the incidence but also greatly increases the progression of skin cancers associated with ultraviolet irradiation (Rossman et al. 2004). Furthermore, transplacental exposure to arsenic is an effective carcinogen in mice, resulting in malignant tumors of the liver and lung (Waalkes et al. 2004). In this model system, arsenic also appears to act as a tumor progressor because it greatly increases malignant liver tumor multiplicity (Waalkes et al. 2004). Although arsenic exposure is associated with prostate cancer in humans (Chen and Wang, 1990; Lewis et al. 1999), the role of arsenic in prostate cancer progression is undefined.

Prostate cancer is the second leading cause of cancer death in American men (Crawford 2003). The normal prostate gland requires androgen for growth and maintenance of differentiated function and will undergo regression if androgen is withdrawn (Kyprianou and Isaacs 1988). Prostate cancer therapy often involves orchiectomy and pharmacologic intervention to diminishing availability of androgen at the androgen receptor (AR) within prostate cancer cells. However, prostate cancer cells often lose the need for androgen as a survival, growth, or differentiation factor and become androgen independent (Westin and Bergh 1998). Although poorly understood, this progression to androgen independence is clearly a critical step in the development of advanced prostate cancer (Suzuki et al. 2003). Androgen-independent prostate cancers are typically more advanced and difficult to treat, and acquisition of such independence has been called a “death sentence” for prostate cancer patients (Arnold and Isaacs 2002).

Altered AR levels or activity can be key elements in acquired androgen independence in prostate cancer. AR is a nuclear transcription factor that normally binds androgen to activate its signaling pathway. Prostate cancer cells can achieve functional AR signaling in the presence of greatly diminished androgens in a variety of ways (Deutsch et al. 2004). AR gene amplification and overexpression can make cells hypersensitive to low levels of androgen, and many prostate cancers show overexpression of AR (Taplin and Balk 2004; Visakorpi et al. 1995). In addition, AR mutations have been recognized that change the ligand specificity of AR such that it can be activated by nonandrogens and even antiandrogens (Deutsch et al. 2004; Tilley et al. 1996). Furthermore, ligand-independent activation of the AR pathway appears to occur in some instances, creating, in essence, a bypass of AR (Feldman and Feldman 2001; Gleave et al. 1999). For instance, certain growth factors, such as insulin-like growth factor-1, keratinocyte growth factor, and epidermal growth factor (EGF), as well as HER2/ neu, a member of the EGF-receptor family of receptor tyrosine kinase, can activate AR-dependent genes in absence of AR ligand (Culig et al. 1994; Yeh et al. 1999). Thus, evidence suggests that altered AR levels, activity, or function can play a major role in the development of androgen-refractory prostate cancer cells (Deutsch et al. 2004; Zegarra-Moro et al. 2002), although an AR bypass can also be important (Culig et al. 1994; Yeh et al. 1999). In men the primary circulating androgen is testosterone. In the prostate, testosterone is converted to the more potent androgen 5-α-dihydrotestosterone (DHT) by the enzyme 5α-reductase (5α-R) (Bonkhoff et al. 1996). DHT is 3–10 times more potent than testosterone in activating AR-regulated downstream events (Martin and Coffey 1998). There is evidence that a significant portion of human prostate cancers overexpress 5α-R type 1 (Thomas et al. 2003). Androgens may be converted to estrogens by the enzyme 5α-aromatase (5α-A) (Simpson et al. 1999). This aromatase is expressed in the human prostate, suggesting a local role for estrogen. Indeed, estrogen can elicit direct actions affecting the growth of prostate cells and can affect estrogen receptor (ER)-mediated gene transcription (Curtis et al. 1997; Robertson et al. 1996). Estrogens have been implicated in the promotion of aberrant prostate growth (Farnsworth 1999) and do not necessarily always work through indirect inhibition of androgen pathways (Harkonen and Makela 2004). In animal models, it has been well established that estrogen may play an important role in prostate carcinogenesis (Bosland 2000). As in other tissues, the effects of estrogen on the prostate are likely transduced primarily by ERs. Prostate cells can be a direct target of estrogen regulation, because they contain both ER-α and ER-β(Harkonen and Makela 2004). Recent evidence indicates that antiestrogens can perturb prostate cancer formation and progression and that this effect is at the level of the ER within prostate cells (Harkonen and Makela 2004; Raghow et al. 2002).

In the present study, we used a model system in which chronic arsenic exposure induced malignant transformation of the human prostate epithelial cell line RWPE-1 (Achanzar et al. 2002) in order to help define the role of arsenic in prostate cancer progression. These transformed CAsE-PE cells rapidly produce very aggressive prostate carcinoma-like tumors upon inoculation into nude mice that overexpress prostate-specific antigen (PSA) while maintaining epithelial characteristics (Achanzar et al. 2002). Specifically, we tested the hypothesis that arsenic may induce androgen-independent growth of human prostate epithelial cells. Our data show that there is loss of androgen dependence after chronic arsenic exposure and the simultaneous acquisition of an aggressive growth behavior. AR expression or ligand specificity played a minimal role in this arsenic-induced prostate cancer cell progression.

## Materials and Methods

### Chemicals and reagents.

We purchased sodium arsenite (NaAsO_2_; purity, 96.6%) from Sigma Chemical Co. (St. Louis, MO) and keratinocyte serum-free medium (K-SFM), EGF, bovine pituitary extract (BPE), 100× antibiotic-antimycotic mixture, and TRIzol reagent from Life Technologies, Inc. (Grand Island, NY). The mouse monoclonal anti-ER-α, the rabbit polyclonal anti-ER-β, and the mouse monoclonal antiactin were purchased from Oncogene Research Products (Cambridge, MA). We purchased the rabbit polyclonal anti-AR from Affinity BioReagents (Golden, CO); horse-radish peroxidase–conjugated secondary antibody from Amersham (Piscataway, NJ); and the Quick Start Bradford protein assay from Bio-Rad Laboratories (Hercules, CA).

### Cells and cell culture.

Control (untransformed) RWPE-1 cells were originally derived from normal human prostate epithelial cells and are immortalized but nontumorigenic (Bello et al. 1997; Webber et al. 1997). Unless otherwise noted, cells were grown in K-SFM containing 50 μg/mL BPE and 5 ng/mL EGF, supplemented with 1% antibiotic/antimycotic mixture. K-SFM containing BPE and EGF is henceforth termed “complete medium.” Cultures were incubated at 37°C in a humidified atmosphere containing 5% CO_2_ and passaged weekly. Cells were exposed continuously to 5 μM arsenite (as NaAsO_2_). The arsenic-exposed cells were designated chronic-arsenic–exposed prostate epithelial (CAsE-PE) cells to distinguish them from the parental RWPE-1 control cells. Parallel cultures grown in arsenic-free medium provided passage-matched controls. After 29 weeks of exposure, CAsE-PE cells produced malignant tumors when inoculated into nude mice (Achanzar et al. 2002). To establish persistence of the observed changes, cells that had been treated for 30 weeks with arsenic were grown in arsenic-free medium for an additional 6 weeks. The phenotypic changes observed in CAsE-PE cells were stable during this period.

### Cell growth rate and the effects of steroids.

To determine the rate of cellular growth, normal and transformed prostate epithelial cells were seeded at a density of 3.2 × 10^3^ cells/cm^2^ in six-well culture plates, and cell proliferation was determined by cell counting as previously described (Igawa et al. 2002). After 3 days, one set of cells was harvested and counted at time 0 with a Z1 model Coulter counter (Coulter Corporation, Miami, FL). The remaining cells were provided with fresh complete medium, and total cell number was determined at various times thereafter. Fresh complete medium was added to the cells every 2 days. Steroid-reduced medium was K-SFM without BPE and EGF. The BPE is likely the major source of steroids in complete medium. To determine the effect of steroid depletion on cell proliferation, cells were exposed to steroid-depleted medium for 2 days, harvested, and counted at time 0. Additional cells were counted on days 3, 6, and 10. Fresh medium was added at each time point. To determine the effects of exogenous androgen or estradiol (E_2_) effects, cells were seeded at a density of 4 × 10^3^ cells/cm^2^ and maintained in regular culture medium for 3 days. Cells were then fed with the steroid-reduced medium and cultured for an additional 48 hr before addition of DHT (0.1 μM) or E_2_ (1 μM; both from Sigma). In a separate series of experiments to test the effects of AR blockade on cell growth, control and CAsE-PE cells were grown in steroid-depleted media for 48 hr then fed fresh steroid-depleted media with or without the antiandrogen flutamide (5μg/mL; Sigma) in the absense or presence of DHT (0.1μM). Cell proliferation was then determined after an additional 4 days. Cells were harvested at various time periods after treatment, and cell numbers were determined.

### RNA extraction and RT-PCR.

Total RNA was isolated using TRIzol reagent by manufacturer’s instructions. Reverse transcription–polymerase chain reaction (RT-PCR) was performed using a TITANIUM one-step RT-PCR kit (Clontech, San Jose, CA) and a GeneAmp PCR system 9700 (Applied Biosystems, Foster City, CA) according to the kit’s instructions. Amplification conditions were as follows: 60 min at 50°C and 5 min at 94°C followed by 35 cycles for 1 sec at 94°C, 1 sec at 55°C (ER-α), 58°C (ER-β), 50°C (5α-A and 5α-R), or 54°C (PSA), 1 min at 72°C; 1 μg total RNA was used in each amplification. Primers were designed for ER-α, ER-β, 5α-A, 5α-R, PSA, and β-actin and were synthesized by Invitrogen (Grand Island, NY) as follows: ER-α(5′-TACTGCATCAGATCCAAGGG-3′ and 5′-ATCAATGGTGCACTGGTTGG-3′), product size: 650 bp; ERβ(5′-TGAAAAGGAAGGTTAGTGGGAACC-3′ and 3′-TGGTCAGGGACATCATCATGG-5′), product size: 530 bp; 5α-A (5′-ATACCAGGTCCTGGCTACTG-3′ and 5′-TTGTTGTTAAATATGATGCC-3′), product size: 273 bp; 5α-R1 (5′-AGCAGATACTTGAGCCA-3′ and 5′-CCAAAATAGTTGGCTGC-3′), product size: 209 bp; 5α-R2 (5′-ACATTACTTCCACAGGACATTT-3′ and 5′-AGGAAATTGGCTCCAGA-3′), product size: 318 bp; PSA (5′-GAGGTCCACACACTGAAGTT-3′ and 5′-CCTCCTGAAGAATCGATTCCT-3′), product size: 214 bp; β-actin (5′-AGAGATGGCCACGGCTGCTT-3′ and 5′-ATTTGCGGTGGACGATGGAG-3′), product size: 460 bp. PCR products were electrophoresed on 1.7% agarose gels, and the gel image was captured and quantified with a Gel Doc 2000 System equipped with TDS Quantity One software (Bio-Rad). The level of β-actin was used to normalize results.

### Western blot analysis.

Total proteins were isolated using M-PER reagent (Pierce, Rockford, IL) as directed by the manufacturer. Protein concentration was determined using the Bradford assay, and 20–40 μg of each sample was electrophoresed on NuPage 4–12% Bis-Tris gels (200V, 30 min) and transferred to nitrocellulose membranes according to the manufacturer’s directions (Invitrogen). Immunoblotting was performed using the ER-αantibody at a 1:100 dilution, horseradish peroxidase–conjugated anti-mouse secondary antibody at a 1:5,000 dilution, ER-βantibody at a 1:1,000 dilution, AR antibody at a 1:100 dilution, or horseradish peroxidase–conjugated anti-rabbit secondary antibody at a 1:5,000 dilution, and SuperSignal West Pico chemiluminescent substrate (Pierce). Signals were visualized by exposure to Hyperfilm (Amersham). Densitometric analysis was performed using Quantity One software (Bio-Rad). AR levels were assessed with and without treatment with the nonmetabolizable androgen mibolerone (5 nM, 6 days; Sigma).

### Statistical analysis.

All data are represented as mean ± SE derived from three or more independent experiments. Statistical significance of the results was determined by the Student’s *t*-test or analysis of variance followed by Dunnett’s *t*-test as appropriate, with *p* ≤0.05 considered statistically significant.

## Results

### Impact of arsenic-induced malignant transformation on cellular proliferation.

**Figure 1:**
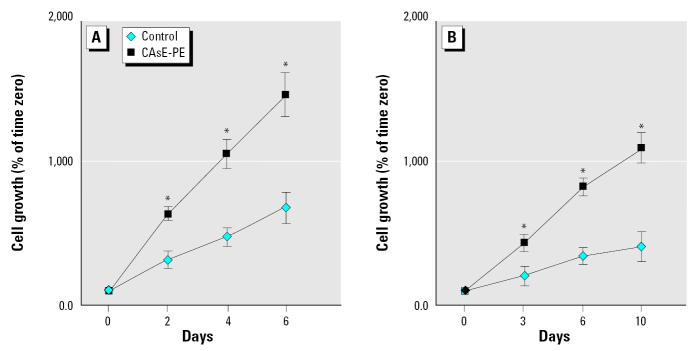
Growth rates of control (RWPE-1) and CAsE-PE cells in complete (*A*) and steroid-reduced (*B*) medium. Cells were seeded and maintained as described in “Materials and Methods.” The total cell number was counted on days 2, 4, and 6 for cells grown in complete culture medium (*A*) or on days 3, 6, and 10 in steroid-reduced medium (*B*). The data shown are the means of triplicate wells after normalization to day 0, indicating cell growth (*n* = 3); error bars represent SE. *Significantly different from control at the same time point.

Arsenic can induce malignant transformation of the human prostate epithelial cell line RWPE-1, such that the transformed CAsE-PE cell line produces aggressive tumors remarkably resembling prostate carcinoma upon inoculation into nude mice (Achanzar et al. 2002). Because androgen independence is often associated with advanced prostate cancers, we examined the growth of control and arsenic-transformed prostate epithelial cells in complete or steroid-reduced medium. In complete medium, the transformed CAsE-PE cells proliferated approximately twice as fast as control cells (Figure 1A), in keeping with their malignant behavior. In a steroid-reduced medium (K-SFM medium without steroid-containing BPE complement or EGF), the growth rate of both cell lines decreased (Figure 1B). However, CAsE-PE cells still had a much more rapid growth rate in steroid-depleted medium, with a doubling time approximately 2.5-fold higher than control cells. Thus, the transformed CAsE-PE cells showed a more rapid growth than did control cells, which was at least partially independent of exogenous steroids. This is consistent with androgen independence in CAsE-PE cells.

Among many possible mechanisms, there are four ways by which androgen independence is attained in prostate cancers through modification of the AR status or function: *a*) overexpression of functional AR, *b*) AR mutation resulting in hyper-responsiveness to androgens, *c*) activation by nonandrogens (loss of ligand specificity), or *d*) activation of ligand-independent AR signaling pathways (Deutsch et al. 2004). Thus, experiments were designed to test these possibilities.

### AR expression, responsiveness, and activity.

**Figure 2:**
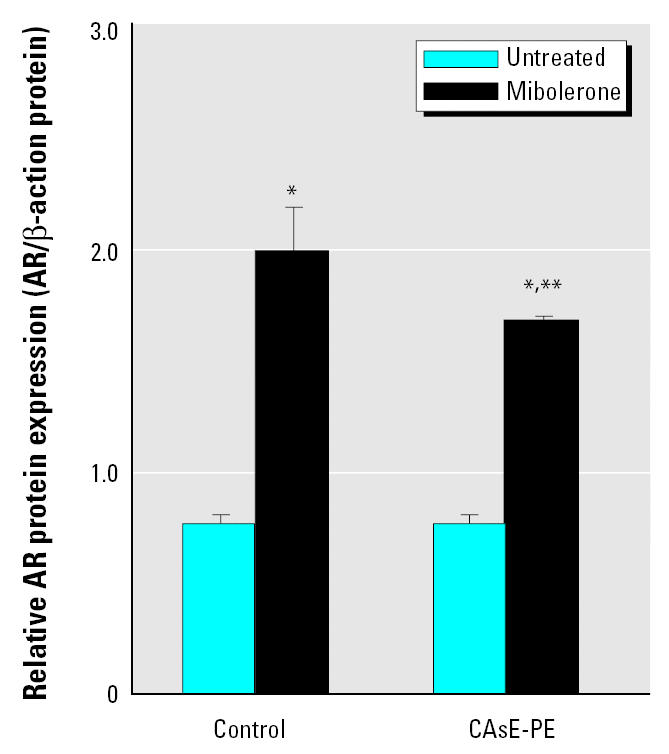
Basal and mibolerone-induced AR protein expression in control (RWPE-1) and CAsE-PE cells assessed by Western blot analysis. Cells were grown in complete medium, and mibolerone (5 nM) was added 6 days before assessment. Densitometric data normalized to β-actin are given as fold increase over control and are expressed as means (*n* = 3); error bars represent SE. *Significantly different from untreated cell-line–matched cells. **Significantly different from control cells treated with mibolerone.

To determine whether the androgen-independent growth in CAsE-PE cells was dictated by overexpression of AR, we conducted AR expression analysis. As shown in Figure 2, AR protein in both control and CAsE-PE cells was expressed at the same level. Thus, overexpression was clearly not required for the apparent steroid-independent growth in CAsE-PE cells. Other studies have shown that androgens can increase AR levels via up-regulation of AR (Yeap et al. 1999). To help test AR responsiveness in control and CAsE-PE cells, mibolerone, a nonmetabolizable androgen, was used to induce AR expression. Mibolerone produced a 2.6-fold increase in the AR protein level in control cells but increased AR in CAsE-PE cells to a significantly lesser extent (Figure 2).

**Figure 3:**
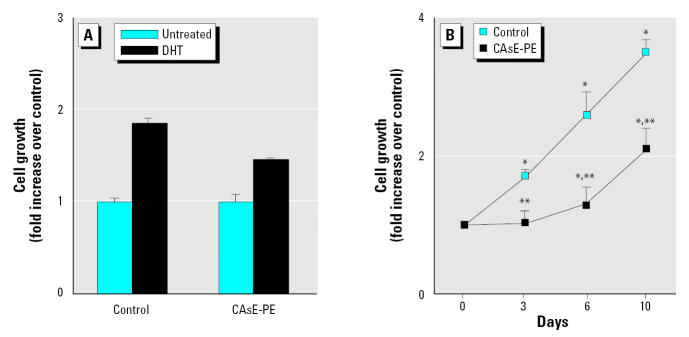
Effect of DHT on the growth of control and CAsE-PE cells. (*A*) Cells were plated in the presence of 0.1 μM DHT, harvested at 7 days, and counted; growth stimulation by DHT was normalized to the control cells (set as 1.0). (*B*) Time course of growth stimulation of normal and arsenic-transformed prostate epithelial cells by 0.1 μM DHT; the total cell numbers were counted on days 3, 6, and 10. Densitometric data are given as fold increase over control and are expressed as means (*n* = 3); error bars represent SE. *Significantly different from untreated, cell-line–matched cells. **Significantly different from control cells treated with DHT.

DHT is known to stimulate gene expression and prostate cell growth through AR. When DHT was added to cells growing in reduced steroid medium, both control and CAsE-PE cells exhibited growth stimulation (Figure 3A). However, the growth of control cells was stimulated nearly 2-fold by DHT at optimal levels (0.1 μM), whereas arsenic-transformed CAsE-PE cells showed significantly less growth stimulation (Figure 3A). The time course for DHT stimulation of cellular growth of control and CAsE-PE cells clearly shows the diminished response in CAsE-PE cells (Figure 3B). The growth of control cells on day 10 was stimulated by DHT approximately 3.5-fold, whereas the growth of CAsE-PE cells was increased only about 2-fold compared with cells grown in steroid-depleted medium. Thus, arsenic-induced malignant transformation actually appears to confer a diminished responsiveness of AR.

**Figure 4:**
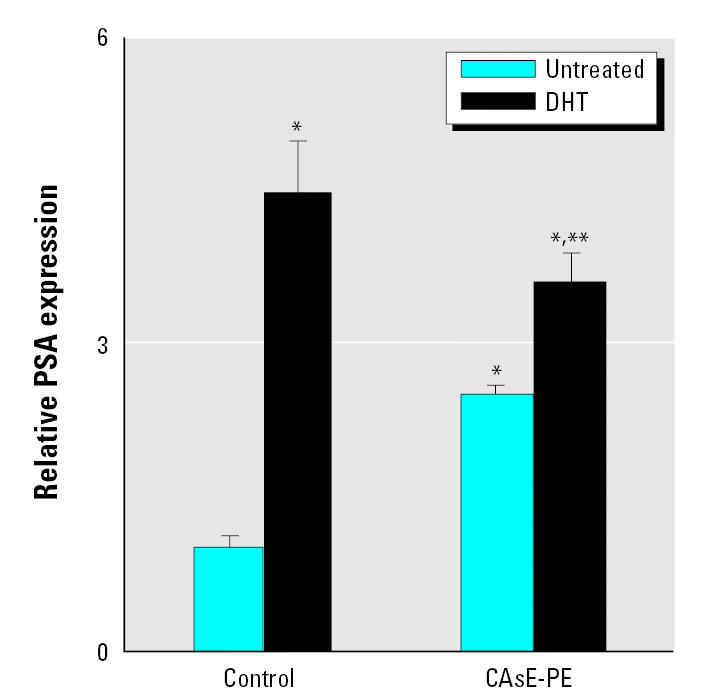
Androgen effects on PSA expression of control (RWPE-1) and CAsE-PE cells. RNA was isolated and subjected to RT-PCR analysis using a set of primers designed to amplify PSA and β-actin gene products after DHT treatment. See “Material and Methods” for details. *Significantly different from untreated control cells. **Significantly different from control cells treated with DHT.

To further assess the activity of AR in these cells, we examined androgen-induced gene expression through AR stimulation. In this case, we examined PSA expression, which is activated by androgens through AR. As is typical with prostate malignancies, CAsE-PE cells expressed significantly more PSA than did control cells (Figure 4). However, a marked 4.6-fold increase in cellular PSA occurred with DHT treatment in control cells, whereas levels increased only 23% in CAsE-PE cells. Indeed, DHT-induced increases in PSA were to a significantly lower maximal level in CAsE-PE cells compared with control cells (Figure 4). This indicates that stimulation of the AR pathway by androgen is less effective in production of AR-related products in arsenic-transformed cells. These data, together with mibolerone data, indicate that the AR in CAsE-PE cells is actually less responsive, and argue against an AR mutation that causes AR hypersensitivity to androgens in these cells.

### Impact of antiandrogens on cell proliferation.

**Figure 5:**
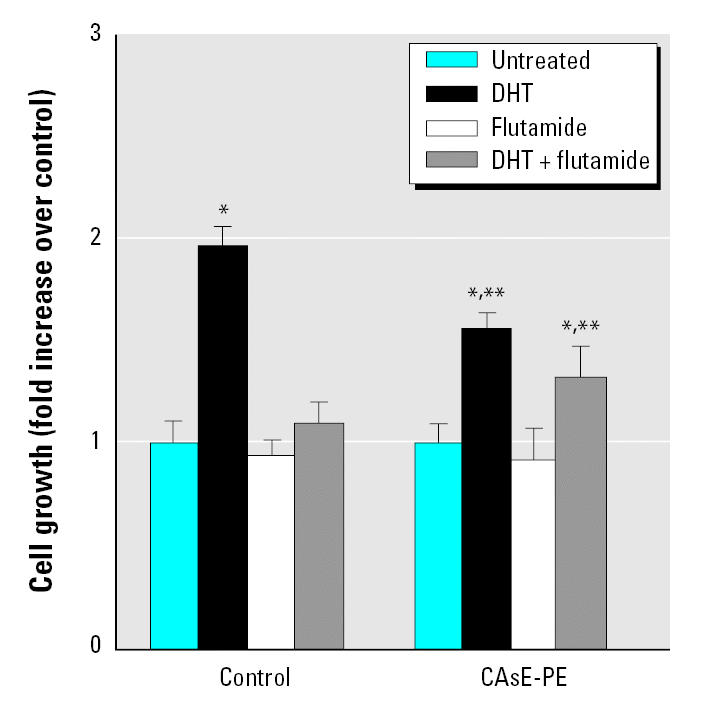
The effect of flutamide on the growth of control (RWPE-1) and CAsE-PE cells. Control and CAsE-PE were exposed to flutamide in the presence or absence of DHT. Data are expressed as means (*n* = 3); error bars represent SE. *Significantly different from untreated, cell-line–matched cells. **Significantly different from control cells treated with DHT or DHT plus flutamide.

Because androgen-independent prostate cancers often become resistant to antiandrogens and AR mutations can result in stimulation by other steroids, including antiandrogens, we tested the effect of the antiandrogen flutamide on DHT-stimulated growth in control and CAsE-PE cells. DHT-stimulated growth was completely suppressed by flutamide in control cells (Figure 5). On the other hand, in CAsE-PE cells, the androgen-stimulated growth was blocked only partially by flutamide.

Collectively, CAsE-PE cells responded differently to DHT, flutamide, or mibolerone, all of which are thought to act through the AR. In light of the findings that AR levels are similar, the androgen-independent growth component of CAsE-PE cells does not appear to be due to overexpression of a functional AR, or through an AR modification that alters steroid sensitivity or selectivity.

### Expression of androgen metabolism enzymes.

**Figure 6:**
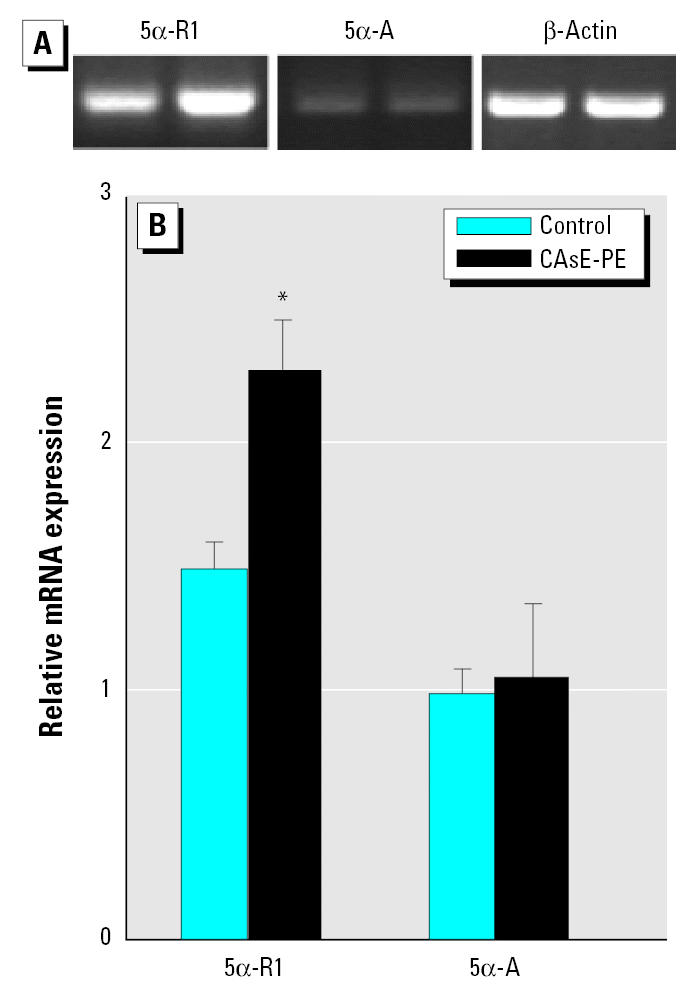
Expression of 5α-R1 and 5α-A in control (RWPE-1) and CAsE-PE cells. RNA was isolated and subjected to RT-PCR analysis using a set of primers designed to amplify 5α-R, 5α-A, and β-actin genes products. (*A*) Representative blot. (*B*) Densitometric analysis normalized to β-actin. Data are expressed as means (*n* = 3); error bars represent SE. *Significantly different from control cells.

It is possible that an aspect of androgen independence in CAsE-PE cells could involve a more efficient conversion of testosterone to DHT by 5α-R. Thus, we evaluated the expression of 5α-R isoforms in control and CAsE-PE cells (Figure 6). Both control and arsenic-transformed CAsE-PE cells expressed 5α-R1 RNA with an elevated expression in CAsE-PE cells (~ 53%). 5α-R2 mRNA was not detectable in either control or CAsE-PE cells (data not shown). The expression of 5α-A, which produces E_2_ from testosterone, was also assessed in each cell line, and both cell lines showed a similar expression level.

### Effect of E_2_ on cell proliferation.

**Figure 7:**
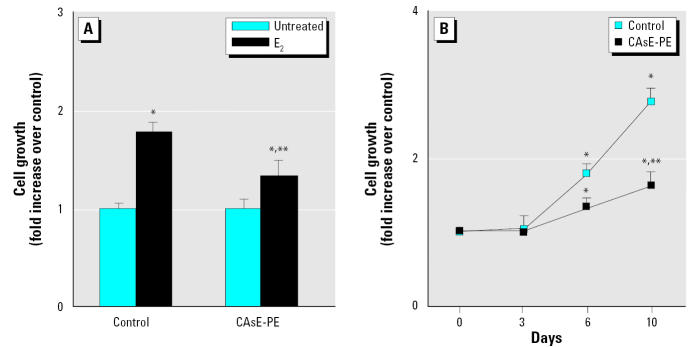
Effect of E_2_ on the growth of control (RWPE-1) and CAsE-PE cells plated in the presence of 1 μM E_2_, harvested at 7 days, and counted. (*A*) Growth stimulation by E_2_ normalized to the control cells (set as 1.0). (*B*) Time course of growth stimulation of normal and arsenic-transformed prostate epithelial cells by 1 μM E_2_. Total cell numbers were counted on days 3, 6, and 10; the data shown are the means and SEs of triplicates. Similar results were found in two independent experiments. Densitometric data are given as fold increase over control and are expressed as means (*n* = 3); error bars represent SE. *Significantly different from untreated cell-line–matched cells. **Significantly different from control cells treated with E_2_.

In many instances of acquired androgen independence in prostate cancer, the AR is modified such that it becomes sensitive to a variety of steroids, including nonandrogens. To test this hypothesis, we also determined the cellular growth of control and CAsE-PE cells after exposure to various concentrations of E_2_. Both control and CAsE-PE cells exhibited optimal growth stimulation by E_2_ at a concentration of 1 μM (Figure 7A). However, the growth of control cells on day 7 was stimulated by 1.8-fold, whereas the growth of CAsE-PE cells was stimulated only about 1.2-fold. We subsequently determined the time course of cellular growth of control and CAsE-PE cells after exposure to 1 μM E_2_. As shown in Figure 7B, the growth of control cells on day 10 was stimulated by approximately 3-fold, whereas the growth of CAsE-PE cells was increased only about 1.3-fold. Growth of control cells is significantly stimulated by physiologic concentrations of E_2_; this growth increase appears to be comparable with that induced by DHT. In contrast, the E_2_ growth-stimulating effect in CAsE-PE cells is significantly less than that observed in control cells.

**Figure 8:**
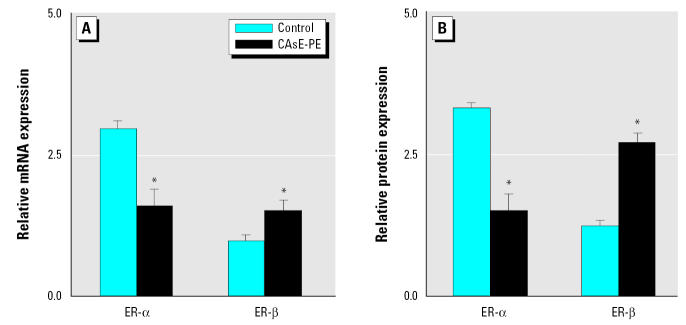
Expression of ERs in control (RWPE-1) and CAsE-PE cells. (*A*) RNA was isolated and subjected to RT-PCR analysis using a set of primers designed to amplify ER-α, ER-β, and β-actin gene products. (*B*) Proteins were isolated and separated and subjected to Western blot analysis monoclonal anti-ER-α, polyclonal anti-ER-β, and monoclonal antiactin. Densitometric data are normalized to β-actin and expressed as means (*n* = 3); error bars represent SE. *Significantly different from control cells.

We also studied the expression of ERs. ER-αand ER-βtranscripts occurred in both control and CAsE-PE cells (Figure 8A). ER-α was down-regulated in CAsE-PE cells compared with control cells (~ 50%, *p* < 0.05). ER-βmRNA levels are distinctly lower in both cell lines compared with ER-α. Nevertheless, ER-βexpression was increased in CAsE-PE cells compared with control cells (1.6-fold, *p* = 0.05). ER-αand ER-βproteins were expressed in both control and CAsE-PE cells and were consistent with the data on mRNA (Figure 8B).

## Discussion

The results demonstrate that inorganic arsenic can potentially affect prostate cancer progression. In this regard, a clear transition from the androgen-sensitive to androgen-independent state occurs during arsenic-induced malignant transformation of human prostate epithelial cells. The androgen response program is critical to the progression of human prostate cancer (Feldman and Feldman 2001). Prostate cancer initially requires androgen for growth and responds to hormone ablation therapies (Feldman and Feldman 2001). However, the disease often progresses to a state of reduced hormone dependence, which is commonly fatal (Arnold and Isaacs 2002). Several mechanisms may contribute to the progression of prostate cancer to an androgen-independent state (Grossmann et al. 2001). AR amplification is found in approximately 30% of clinically advanced prostate cancer cases (Koivisto et al. 1997; Visakorpi et al. 1995). Overexpression of transcriptional coactivators also accompanies progression in some cases and facilitates the activity of AR (Comuzzi et al. 2003). Mutations in the AR may allow it to respond to different steroids as well as antiandrogens (Taplin et al. 1999). However, arsenic-induced androgen independence in CAsE-PE cells is not associated with AR overexpression or altered AR ligand specificity, indicating that arsenic affects progression through a non-AR-dependent mechanism. In this regard, the growth factors and receptors associated with prostate cancer progression often regulate cell growth through stimulation of Ras signaling pathways (Weber and Gioeli 2004). Recent data from our laboratory indicate that wild-type k-*ras* activation is strongly correlated with arsenic-induced transformation in CAsE-PE cells (Benbrahim-Tallaa et al., in press). Chronic activation of *ras* by autocrine and paracrine growth factor stimulation is thought to be a common mechanism for prostate cancer progression, and attenuation of *ras* signaling can restore androgen sensitivity to hormone-refractory prostate cancer cells (Bakin et al. 2003a, 2003b). Because arsenic-induced androgen independence does not appear to involve AR overexpression or altered ligand specificity, a bypass of AR through chronic overexpression of Ras may well contribute to this progression. Further research on arsenic stimulation of this important growth signaling pathway is ongoing.

The role of ER in prostate cancer progression is not completely understood. In the present study, ER-αexpression was significantly reduced in arsenic-transformed CAsE-PE cells. ER-αexpression is often down-regulated in prostate cancer (Linja et al. 2003) and is associated with a poor prognosis because it reduces the effectiveness of endocrine therapy (Konishi et al. 1993). Thus, the reduced ER-αexpression in arsenic-transformed CAsE-PE cells may indicate a more advanced tumor cell, consistent with the production of invasive carcinoma when these cells are inoculated into nude mice (Achanzar et al. 2002). ER-βexpression may be reduced in primary prostate cancers, but its expression returns in metastases (Weihua et al. 2002). In fact, recent studies have shown that ER-βis the predominant ER subtype expressed in prostate cancer metastases (Lai et al. 2004; Leav et al. 2001). Therefore, the overexpression of ER-βin CAsE-PE cells may also suggest a more progressed state.

Both control and CAsE-PE cells expressed only the type 1 isoform of 5α-R in the present study. This is consistent with the androgen-independent prostate tumor cell lines DU-145 and PC3 (Delos et al. 1995; Negri-Cesi et al. 1999) and isolated human prostate cancer epithelial cells (Delos et al. 1995) where only 5α-R1 is detected. Generally speaking, 5α-R1 appears to predominate in cancerous prostate tissue (Occhiato et al. 2004) and is highly overexpressed in a subset of prostate cancers but not highly expressed in benign prostatic hyperplasia (Thomas et al. 2003). The overexpression of 5α-R1 in arsenic-transformed CAsE-PE cells indicates that it is possible that these cells could convert more testosterone to DHT. However, 5α-R1 overexpression does not appear to be involved in aberrant cell proliferation in DU-145 cells, because a specific 5α-R1 inhibitor (LY306089), which blocks DHT formation, has no effect on proliferation of DU-145 cells (Kaefer et al. 1996). Thus, 5α-R1 overexpression does not appear to account for the hyperproliferation observed for arsenic-transformed CAsE-PE cells.

The low levels of 5α-A transcript in control and CAsE-PE cells indicate that the intracellular production of estrogens is not a characteristic of these cells and limits the possibility that testosterone, through estrogen formation, might still indirectly be active in CAsE-PE cells. 5α-A is observed in normal and pathologic prostate specimens (Matzkin and Soloway 1992) and in prostate cancer cells (Block et al. 1996). However, the poor response to E_2_ and the very weak expression of aromatase in androgen-independent CAsE-PE cells indicates local aromatization of testosterone probably does not play a major role in arsenic-induced prostate cancer progression.

In summary, the present results clearly show that arsenic can precipitate events leading to rapid growth and greatly reduce androgen dependence during malignant transformation of human prostate epithelial cells. Arsenic-induced acquisition of androgen independence does not involve overexpression of AR or any apparent changes in AR ligand sensitivity. Changes in androgen metabolism, estrogen production, or ER levels and sensitivity also appear to have limited roles in this conversion. However, the fact that a common contaminant of the human environment can potentially affect prostate cancer progression provides strong incentive to further define the role of arsenic in prostate cancer progression.
